# Non-Invasive Detection of Early Retinal Neuronal Degeneration by Ultrahigh Resolution Optical Coherence Tomography

**DOI:** 10.1371/journal.pone.0093916

**Published:** 2014-04-28

**Authors:** Debbie Tudor, Vedran Kajić, Sara Rey, Irina Erchova, Boris Považay, Bernd Hofer, Kate A. Powell, David Marshall, Paul L. Rosin, Wolfgang Drexler, James E. Morgan

**Affiliations:** 1 School of Optometry and Vision Sciences, Cardiff University, Cardiff, United Kingdom; 2 School of Computer Science and Informatics, Cardiff University, Cardiff, United Kingdom; 3 School of Biosciences, Cardiff University, Cardiff, United Kingdom; 4 Center for Medical Physics and Biomedical Engineering, Medical University Vienna, Vienna, Austria; 5 Department of Neurology and Ophthalmology, School of Medicine, Cardiff University, Cardiff, United Kingdom; Justus-Liebig-University Giessen, Germany

## Abstract

Optical coherence tomography (OCT) has revolutionises the diagnosis of retinal disease based on the detection of microscopic rather than subcellular changes in retinal anatomy. However, currently the technique is limited to the detection of microscopic rather than subcellular changes in retinal anatomy. However, coherence based imaging is extremely sensitive to both changes in optical contrast and cellular events at the micrometer scale, and can generate subtle changes in the spectral content of the OCT image. Here we test the hypothesis that OCT image speckle (image texture) contains information regarding otherwise unresolvable features such as organelle changes arising in the early stages of neuronal degeneration. Using ultrahigh resolution (UHR) OCT imaging at 800 nm (spectral width 140 nm) we developed a robust method of OCT image analyses, based on spatial wavelet and texture-based parameterisation of the image speckle pattern. For the first time we show that this approach allows the non-invasive detection and quantification of early apoptotic changes in neurons within 30 min of neuronal trauma sufficient to result in apoptosis. We show a positive correlation between immunofluorescent labelling of mitochondria (a potential source of changes in cellular optical contrast) with changes in the texture of the OCT images of cultured neurons. Moreover, similar changes in optical contrast were also seen in the retinal ganglion cell- inner plexiform layer in retinal explants following optic nerve transection. The optical clarity of the explants was maintained throughout in the absence of histologically detectable change. Our data suggest that UHR OCT can be used for the non-invasive quantitative assessment of neuronal health, with a particular application to the assessment of early retinal disease.

## Introduction

The quantification of cellular health is essential to allow accurate monitoring of responses to experimental or therapeutic interventions. Many of the available optical techniques to probe cellular health rely on either the use of fluorophores to tag molecules of interest [1], [2] or are limited by the need for a high numerical aperture (NA) in microscopy or Raman spectroscopy [3]. While ligand-based methods can, in vitro, provide robust staging of cellular events, such as apoptosis, the in vivo use of these methods is problematic due to ligand toxicity, the need for ligand delivery, and an inherent bias towards the detection of late stage apoptotic events [4].

The development of a high resolution, non-invasive label-free technique would significantly advance our ability to follow cellular events over time. Interference based imaging technology such as optical coherence tomography (OCT) is particularly promising in this respect and has recently been used for quantification of cell death in vitro and in vivo [5]. Since the axial resolution in OCT is not constrained by the numerical aperture of the focusing optics [6] it has the potential to detect subcellular changes (such as organelle disruption) that are known to precede cell death [7], [8], allowing the non-invasive characterization of early apoptotic events. The availability of broad spectral bandwidth light sources (spectral spread 140 nm and above) has increased the resolution of these devices to 1–2 µm which should, in principle, allow the detection of optical changes driven by organelle alterations at a subcellular scale, even when the identification of individual organelles is not possible.

To explore this possibility, we imaged cultured neurons and retinal explants following the initiation of apoptosis using a specially developed novel spectral domain UHR-OCT. The chosen wavelength (800 nm) permitted the detection of structural changes in the 1 µm–4 µm range [9] that might arise from subcellular organelles (mitochondria, endoplasmic reticulum, Golgi apparatus and the nucleus) which undergo degenerative changes in the early stages of apoptosis. At this wavelength, multiple light scattering produced by these structures [10] will be close to maximal and within the detection limits of UHR-OCT [11]. We therefore reasoned that changes in the light scattering properties of cells in the early stages of apoptosis could be detectable by UHR-OCT at the level of cell populations. The mitochondrial contribution to cellular light scattering [12], [13], if detected, could also be used as a robust biological marker of early neuronal degeneration, as reconfiguration of the mitochondrial network is associated with increased mitochondrial membrane permeability allowing the release of pro-apoptotic molecules such as cytochrome c, and the activation of the caspase cascade [14], [15].

## Results

### Optical detection of apoptosis in cultured neurons

**Figure 1:**
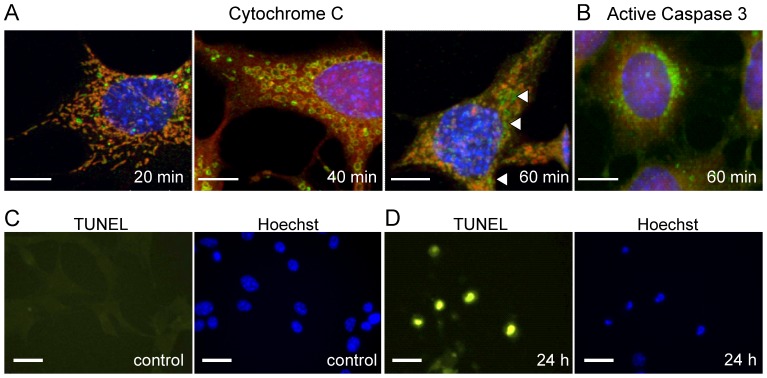
Immunocytochemistry of apoptotic RGC-5 cells. A–B RGC-5 cells during the first hour after induction of apoptosis. Scale bars 100 µm. Cells were pre-treated with CMxRos to identify mitochondrial morphology (red) before probing with antibodies against cytochrome c (green) (**A**) and activated caspase 3 (green) (**B**) as a marker of apoptosis. The arrow heads (**A, third panel**) are pointing to the positive staining for cytochrome c that has leaked out from the mitochondria and into the cell cytoplasm, indicating break down of the mitochondrial membranes. Activation of caspase-3 is an early indicator of apoptosis and is first present at 60 min (**B**). **C–D** RGC-5 cells before and 24 h after induction of apoptosis. Scale bars 20 µm. Apoptotic cells are visualized using green TUNEL staining (left). The cell nuclei are counterstained using Hoechst (blue, right panel). Absence of any green TUNEL staining on the left (**C**) indicates absence of apoptotic cells in control culture. Scale bars 20 µm. Prominent green TUNEL staining (**D**) and reduced size of the nuclei on the Hoechst staining (right) mark apoptotic cells.

We first evaluated the time course of neuronal degeneration and apoptosis in RGC-5 cell cultures following the administration of staurosporine. Structural changes were observed 20 minutes after application as the mitochondria changed from a filamentous network [16] to one composed of discrete rounded structures (Fig. 1A). Mitochondrial cytochrome c release (indicating the break down of the cell membrane [14], [15]) could be detected only 60 minutes after the treatment. These data confirmed the existence of a time window for investigation between the initial changes in mitochondrial morphology and mitochondrial membrane breakdown in which the cells were stressed but maintained functionality. The identification of active caspase 3 at 60 min (Fig. 1B) confirmed that the cells were undergoing apoptosis rather than necrosis. The final stages of apoptosis were identified using TUNEL labeling with over 90% of cells being TUNEL positive 24 hours after the administration of staurosporine (Fig. 1C).

**Figure 2:**
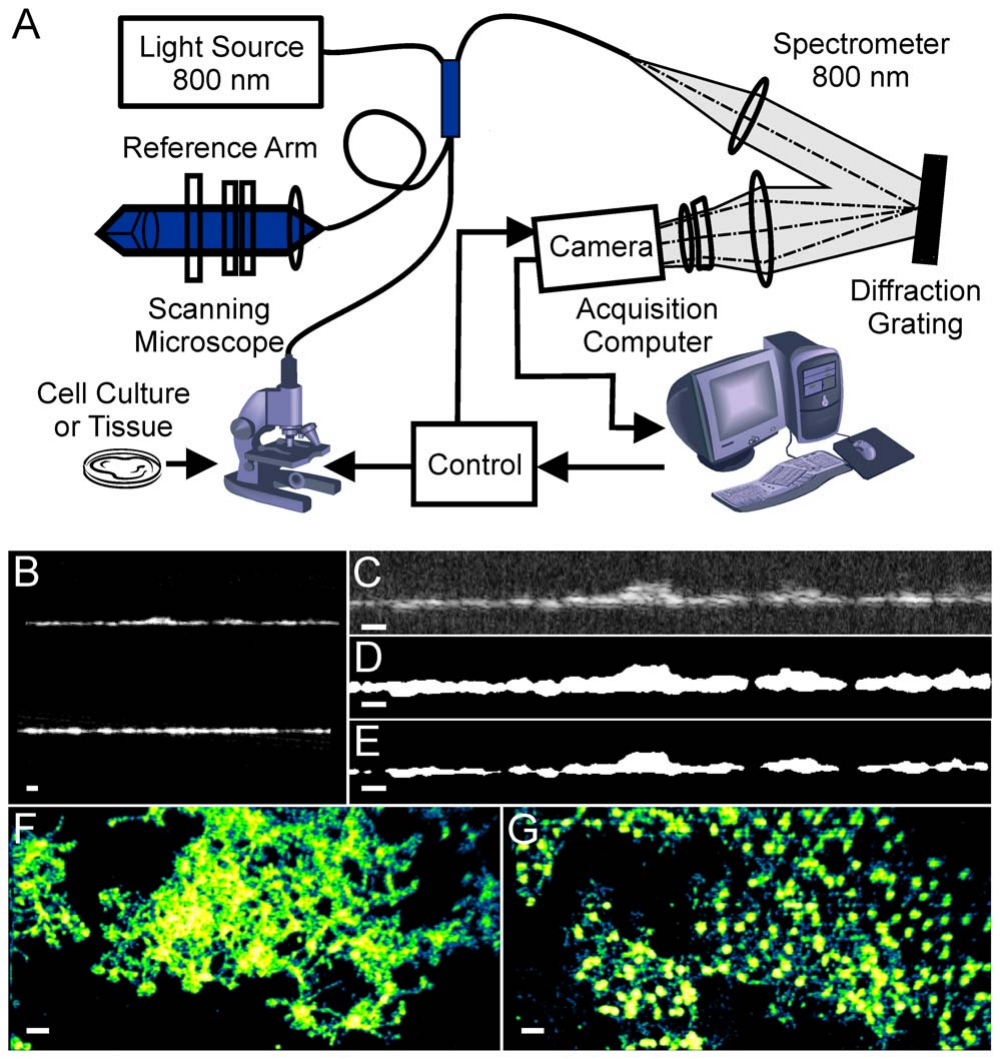
Imaging methodology. A. Schematic diagram of the UHR-OCT system. B–E. Examples of UHR-OCT images of RGC-5 cells; B. Original image; C. Cropped image; D. Processed image with the region of interest masked; E. Refined region of interest; F–G. Post-processed en face images of RGC-5 cells within region of interest in control (F) and apoptotic cell cultures (G). Scale bars 40 µm.

**Figure 3:**
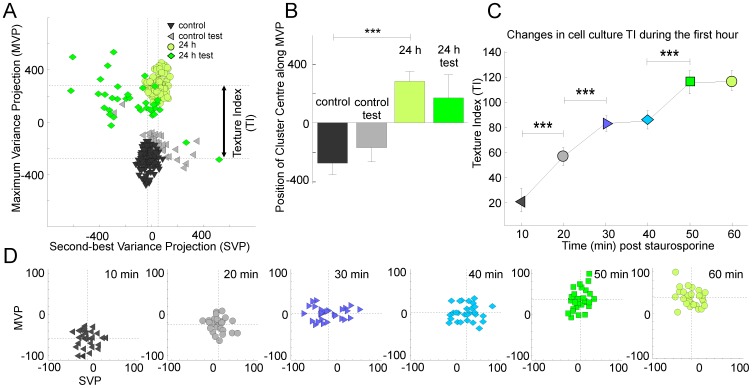
Optical signatures of apoptotic and pre-apoptotic cells. A–B. Gaussian mixture model analysis of control and apoptotic RGC-5 cell images (24 h after treatment with 1 µM Staurosporine). Data were collected from 20 coverslips with 10 images per coverslip taken from different regions within each coverslip. Two distinct classes were identified based on texture analyses (control training set vs 24 h training set). Multidimensional data were visualized using Sammon projection along the axis with the largest (maximum) variance (MVP) and the second best variance (SVP). An automatic procedure placed new images (control test set and 24 h test set) into the correct category in 95% of cases. The Texture Index (TI) was defined as a distance between data classes along the MVP axis. C–D. Gaussian mixture model analysis of early stages of apoptosis. Data were collected from 28 cover slips, imaged every 10 min following the administration of staurosporine. Classes of cells for each time point were identified based on the texture analyses. A progressive shift in Texture Index (TI) defined as before was observed within 20 min of treatment. Significance levels were calculated by t-test (*p<0.05, ** p<0.01,*** p<0.001).

We then determined whether it was possible to distinguish healthy cells from final stage apoptotic cells based exclusively on OCT images captured 24 hours after the instillation of staurosporine (see Fig. 2 and Methods). Data were collected from 20 apoptotic and 20 control RGC-5 cover slip cultures each imaged at 10 discrete locations. Images were processed to remove background noise, detect coverslip position, and remove any tilt and imaging artifacts (Fig. 2B). The part of the image containing the majority of cells (Region of Interest, ROI) was detected automatically based on coverslip position and surrounding pixel intensities (Fig. 2C–E). Each ROI was described by a relatively large set of parameters (65 in total) in an attempt to capture the essence of the image texture. We then combined data from the large number of images of healthy cells to generate a quantitative multidimensional multi-Gaussian representation of the healthy cell population in the selected feature space. Each new image (a new point in the feature space) was characterized by its position in the feature space and by its distance [17] from the center of cluster(s) representing images of healthy cells. We used a Sammon projection method [18] to visualize the distance in multidimensional space [17] as a two dimensional plot. Figure 3A shows that two distinct classes corresponding to healthy and apoptotic cells were clearly visible 24 hours after the administration of staurosporine. When viewed in 2 dimensions, the difference was mainly confined to the projection [18] taken in the direction of the maximum variance (MVP). We therefore selected this projection for subsequent analyses. Figure 3B highlights the difference in the position of the centers of clusters representing images of healthy and apoptotic cell cultures using this metric. To establish whether apoptosis could be detected automatically, we then selected a random subset from each group (test data that were not used for the initial definition of the clusters) to classify images using pattern recognition techniques [19], [20] into either of the two groups. On the basis of this analysis, apoptotic cell cultures could be discriminated automatically with an accuracy of 95% (Fig. 3A,B; test data).

Our immunohistochemical staining (Fig. 1A) indicated that early apoptotic changes could be detected in the first hour following the treatment with staurosporine. We next determined whether OCT could also track the pre-apoptotic optical changes in vitro in the first hour. On the basis of the time course of degenerative changes, cells were imaged at 10 min intervals following the administration of staurosporine. The images were obtained from 34 separate RGC-5 cultures (1 location/culture). OCT signals were processed as for the test cell cultures but the pattern recognition analyses was now limited to a feature space containing only the most informative features [21] (see Methods). To quantify changes we defined a *Texture Index* (TI) as the shift in the position of the center of the cluster projected in the direction of the maximum variance (see Fig. 3A). We observed a progressive shift in the position of the cluster representing treated culture (Fig. 3D), best represented in the growing value of the TI throughout the first hour (Fig. 3C). The most dramatic shift occurred within 30 mins of the administration of staurosporine with δTI approaching 0 at 50 min (Fig. 3C). Algorithms were developed to automate the discrimination process based on this texture analysis; these could separate treated and control cultures imaged at either 0–20 min or 30–60 min after treatment, with an accuracy of 85%. These image data correlated well with changes in mitochondrial labeling and mitochondrial integrity (MitoTracker Red) within 20 minutes of staurosporine administration (Fig. 1A).

### Optical detection of apoptosis in retinal explants

**Figure 4:**
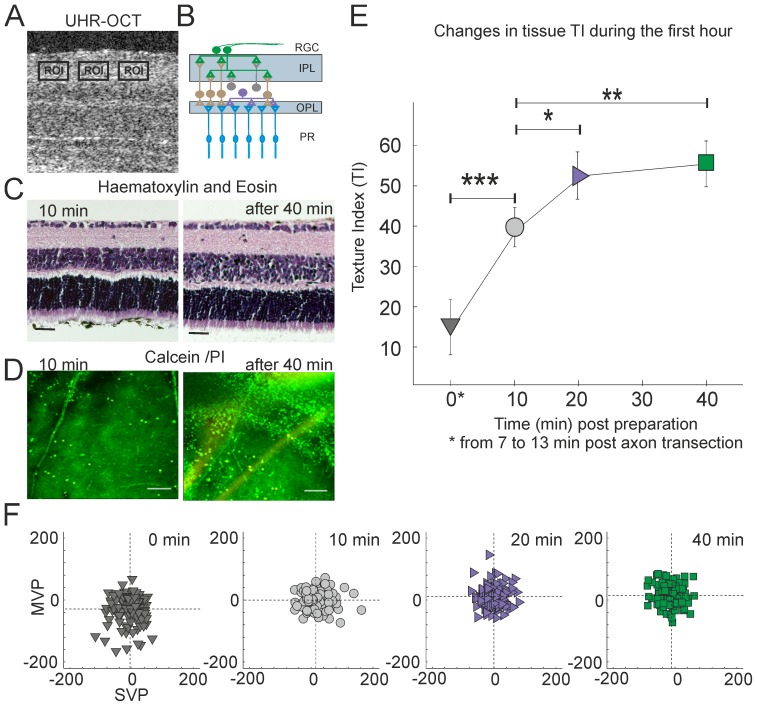
Optical signatures of apoptotic cells in tissue. A–C. Images of murine retinal explants. A: OCT, transverse image through the retina, retinal ganglion cell side up. 10 regions of interest (ROIs) were collected from 13 explants (10 ROI per explants) and imaged every 2 mins during the first hour (imaging started 10–13 min after transection of axons); B.: Schematic drawing of retinal layers (RGC, retinal ganglion cell layer; IPL, inner plexiform layer; OPL, outer plexiform layer; PR, photoreceptor layer). ***C***. The morphology of the explants was preserved during the imaging session as indicate by Haematoxylin and Eosin staining. Scale bars 100 µm. D. Positive Calcein AM (green) but not propidium iodide (PI) staining (red) indicates that the retinal explants remained alive throughout the imaging session. A few dead cells were sometimes observed at the end of imaging session (scale bar 100 mm). E–F. Gaussian mixture model analysis of early apoptosis in retinal explants. A progressive shift (similar to RGC-5 cells) in the Texture Index (TI) was observed over time. Significance levels were calculated by t-test (*p<0.05, ** p<0.01,*** p<0.001).

We then tested whether this method could also be used to quantify neuronal degeneration in intact tissue taking retinal explants (flatmounts, Fig. 4AB) in a model where sectioning of the optic nerve initiated apoptosis in retinal ganglion cells [22]. Preliminary experiments showed that explant viability was critically dependent on sterility, pH, oxygenation and temperature of the culture medium. In general, ex-vivo explants show low number of TUNEL positive cells (less than 5%) up to 7 days in vitro; neuronal death is usually preceded by dendritic degeneration. OCT imaging increased the risk that the explant environment could be compromised resulting in acclerated cell death compared with explants kept in the incubator. However, histological staining (Fig. 4C) indicated that morphology of the explants was preserved throughout the imaging session. Furthermore, we observed positive (green) Calcein staining with no propidium iodide (PI staining, red) throughout the imaging session (Fig. 4D) indicating that the majority of cells remained viable for the duration of the experiment. Since dendritic atrophy is a robust marker for retinal ganglion cell degeneration [23], [24], we manually selected several regions of interest in the inner plexiform layer (IPL) of the retinal explants which contain RGC dendrites rich in mitochondria for the image analyses (Fig. 4A,B, details in Methods) for the quantification of image texture. To avoid areas with prominent blood vessels, several smaller regions of interests within the IPL were manually selected using a custom graphical interface written in Matlab. The feature reduction procedure [21] (Methods) yielded, on average, 15 informative features per image similar to those seen with the RGC-5 cultures. In the first hour following axotomy (Fig. 4E,F) the cluster representing images of axotomised retinal explants shifted in feature space causing the TI to increase monotonically with time. The greatest change in TI occurred within 20 min of optic nerve transection. Our automated discrimination algorithms could sort images taken within first 20 min from those taken 30–60 min post axotomy with 68% accuracy. It is important to note that the retinal explants remained transparent throughout the experiment, indicating that the changes observed were not secondary to opacification of the retinal tissue.

## Discussion

Our study demonstrates that texture analysis of OCT images can be used to generate quantitative surrogate measures of cellular health in the early stages of neuronal degeneration. An important feature of the present study is that we carefully staged the degenerative changes occurring in neuronal populations using in vitro and ex vivo preparations. Automated texture analysis was then able to detect cellular events leading to apoptosis within 30 minutes of the application of staurosporine. While the optical scatter generated by subcellular changes cannot be used directly to quantify the structure of individual organelles it can, however, be used to derive an index characterizing organelle changes in a cell population. Texture analysis therefore represents a significant step forward in the use of OCT for quantifying the integrity of neuronal tissues and, in particularly, the retina.

OCT [25], [26] is acutely sensitive to changes in small-angle forward scatter components in the image arising from subtle variations in cell structure and packing [27]. In the context of quantifying the dimensions of biological structures, this scatter has historically been regarded as a negative influence on image quality [28]. Not surprisingly, commercial OCT systems remove speckle in the image as one of the first processing steps to facilitate the delineation of tissue boundaries [29]. The realization that speckle (coherent noise) contains an optical component arising from the scattering effects of subcellular structures has prompted the use of texture analysis [30], [31] to extract image features that correlate with this signal. Schmitt was among the first to consider the origin of speckle in OCT images, suggesting that it was generated by scattering between photons combined with the interference of wavefronts from multiple scatterers [32]. More recently, there has been increasing interest in the use of speckle information for the extraction of biological information at scales that exceed theoretical resolution of the OCT [33]–[35]. The observation that the optical texture of retinal layers is consistent with their anatomical composition [6] (i.e. whether a given layer is composed predominantly of neurites or cell bodies) indicates that structures beyond the theoretical resolution limit of the OCT can, when viewed as an aggregate structure generate informative textural information. The use of OCT in combination with texture analysis to distinguish differences between similar types of tissues was first described by Gossage et al. [30] who were able, using a combination of statistical and spectral texture analysis, to discriminate tissue types (skin and fat). Texture analysis has also been used with other imaging modalities to characterize tissue at the cellular level. It has been applied to ultrasound images to discriminate choroidal melanoma subtypes with a high degree of diagnostic precision [36]. Texture based measures, obtained by retroillumination methods have also been used to quantify the degree of posterior capsule opacification following cataract surgery [37].

In the present study we have not attempted to identify the source of the scatter. Comparison with cell cultures shows that textural changes occurred at a time when disruption of the mitochondrial network would be expected to occur and we would, on this basis, consider the mitochondrial network as a likely candidate source of the signal changes. In times of cellular stress, mitochondria change from filamentous network in which fused elongated organelles extend over 10 µm [38] to a collection of fragmented rounded organelles [9]. Furthermore, in the closing stages of apoptosis, nuclear structures sized in the range of 3–5 µm [39] should generate additional changes in scatter. Cells texture can also change as a result of the formation of stress granules [40] which form particles in the range 1–2 µm. Significantly, these stress granules can appear and disappear without the initiation of apoptosis and therefore act as a sensitive marker of cellular health [41]. Areas with 1 µm differences in average scatter diameter can be identified/classified using an amplitude and frequency (AM-FM) modulation analysis of the OCT signal to detect subtle changes in the spectral profile resulting from variations in the size of the scatters [42]. Pitris et al used polystyrene beads dispersed in polyacrylamide gels as imaging phantoms to demonstrate a high degree of accuracy [43] in the discrimination of the scatterer particle size.

Our study used stable ex-vivo tissues imaged using high aperture lenses, which may constrain the application of this type of analysis to *in vivo* studies. For the purposes of imaging of living eye where the numerical aperture would be reduced, the techniques should still be transferable, especially in combination with adaptive optics to allow improved transverse resolution [44]. The presence of media opacities such as cataract, while they may reduce the signal to noise ratio, should not prevent high resolution texture analysis since the use of imaging wavelengths greater than 1 µm spacing can limit this type of signal attenuation [45]. Techniques for image stabilization [46], [47] during acquisition and the use of faster cameras should reduce any movement artifacts with in vivo imaging. Since motion artifacts will affect all retinal layers and, on average be similar in eyes regardless of pathological status, these should not be major confounders. We have recently demonstrated that texture analyses could also be employed to enhance the diagnostic capacity of current clinical OCT imaging in the discrimination of eyes with glaucomatous damage [48].

To conclude, we demonstrate for the first time the feasibility of using texture analysis of OCT images for non-invasive characterisation of neuronal (retinal ganglion cell) degeneration. Longer wavelengths, while associated with a slight reduction in axial resolution provide better signal to noise ratio for imaging deeper structures, raising the possibility that texture analysis could also be used for the assessment of the integrity of outer retinal layers.

## Methods

### RGC-5 culture

Cultured RGC-5 cells were obtained from ATCC (American Tissue Culture Collection) and prepared as previously described [49]. Cells were grown on sterile glass coverslips in media containing low glucose DMEM (Sigma) supplemented with 10% FBS (Invitrogen), 4 mM Glutamine (Invitrogen), 100 u/ml penicillin and 100 µg/ml streptomycin (Invitrogen) and incubated at 37°C/5%CO_2_ until 70% confluent. For UHR-OCT imaging, coverslips were put in Sykes-Moore chambers (SciQuip) kept at 37°C with a thermostatically controlled heating stage. To induce apoptosis cells were cultured in serum free growth media containing 1 µM staurosporine.

### Retinal explant culture

C57BL/6 mice (age P15 and above) were obtained from Charles River (UK). All procedures were conducted in accordance with Home Office regulations and the ARVO statement for use of Animals in Ophthalmic and Vision Research. Mice were humanely killed at a designated establishment by CO_2_ induced asphyxiation (Schedule 1 Method, United Kingdom Animal Scientific Procedures Act 1986).The eyes were enucleated and the anterior segment, lens, vitreous body, retinal pigmented epithelium, and sclera removed. The retina was then flat mounted, retinal ganglion side up, on a nitrocellulose insert (Millipore, UK) before being placed in a Sykes-Moore chamber kept at 37°C as for the cell cultures. Explants were held in position using a slice anchor (Harvard Apparatus) and submerged in Neurobasal medium (Invitrogen) supplemented with 2% B27, 0.8 mM L-Glutamine, 15 mM HEPES, 100 U/ml penicillin and 100 mg/ml streptomycin. Explant preparation time was completed in under 13 min in all cases. Explants (n = 13) were then imaged over 60 min, using the same system as for the cell cultures.

### Immunocytochemistry

Mitochondria were labelled using Mitotracker Red (CMxRos) (Invitrogen). RGC-5 cells were treated with 200 nM CMxRos in growth media for 20 min prior to induction of apoptosis. Cells were then fixed at 10 min intervals for the first 60 min and then at 24 h with 1% paraformaldehyde at 4°C overnight.

### Immunohistochemistry

Fixed RGC-5 cells were permeabilised with 0.2% Tween-20 in PBS for 30 min at room temperature before blocking with 5% goat serum in PBS. Cells were incubated with primary antibody directed against the active form of Caspase-3 (Sigma) (1∶100) or cytochrome C (1∶100) overnight at 4°C. Visualisation was achieved by the addition of goat anti rabbit Alexa Fluor 488 for caspase 3 or donkey anti sheep Alexafluor 488 for cytochrome C (Molecular Probes). Cell slides using Prolong Gold antifade reagent (Invitrogen). Caspase 3 labelling was viewed with an epifluorescent microscope (Leica DM6000B). Cytochrome C labelling was imaged with a Leica TCS SP2 AOBS confocal scanning laser microscope (Leica, Heidelberg) using appropriate excitation and emission parameters for Hoechst (Ex. Max: 365; Em Max: 480), Alexa 488 (Ex. Max: 494; Em. Max: 519) and Mitotracker Red (Ex. Max:578; Em. Max: 599). Z-stacks of optical sections were collected through the entire volume of the cells using a z-step of 0.3 µm. Z stacks were viewed as maximum intensity projections prepared using Leica confocal software.

### TUNEL labeling

TUNEL labelling was carried out using the ApopTag Fluorescein in situ apoptosis detection kit (Millipore) as per manufacturer's instructions.

### UHR-OCT system

Images were obtained using a spectrometer-based frequency-domain OCT using a Femtosource Integral OCT Ti: Sapphire laser (Femtolasers GmbH, Austria) with a bandwidth of 140 nm. The sample arm contained a Field Programmable Grid Array (FPGA) integrated with a CameraLink capable frame grabber. The FPGA controlled a galvanometric x/y scanner built into a microscope with a Thorlabs LSM02-BB broadband telecentric scan lens (0.1NA objective, 7.5 mm working distance for cells, 0.06NA and 25.1 mm working distance for explants) (Thorlabs, Newton, New Jersey, USA). The axial resolution was ∼3 µm with the transverse resolution in the range 6–8 µm. The reference arm comprised a moveable stage with a gold-coated hollow corner cube reflector allowing adjustment of the reference delay, and a neutral-density filter-wheel was used to keep the signal just below saturation of the spectrometer (ATMEL AViiVA M2 CL 2048 pixel CCD). The FPGA, real time display and data storage were controlled by custom software written in Labview (National Instruments). The light source was connected to a 90/10 beam splitter (Ipitek Inc, Carlsbad, CA, USA). The scanning microscope was connected to the 10% arm of the beam splitter and samples were exposed to ∼1.4 mW of power only during image acquisition. Images were recorded using a 12 bit 2048 pixel silicon CCD-camera (AVIVA M2 CL2014-BAO, Atmel, CA, USA) at a line rate of 20 kHz and 50 ms exposure time. Sample and reference arm comprised FiberCore SM750 single mode fibers (cut-off wavelength of ∼650 nm, Fibrecore Ltd, UK) which were looped through polarization control paddles to maximise fringe visibility.

### OCT image analyses

OCT images were acquired as stacks of 512 slices sized 512 by 1024 pixels (746 µm by 1492 µm). OCT images were subjected to standard image processing techniques in which the imaging artefacts such as light reflections were removed from the image. The position of the coverslip supporting the cells was detected by examining the direction of high frequency image component (when pixels intensity change rapidly or abruptly), and any tilts in the 3 cardinal directions were removed by rotating the image accordingly. Line scan artefacts were removed by FFT filtering. All image processing was performed in Matlab (Mathworks) using standard functions from image analysis and statistics toolboxes.

### Masking the Region Of Interest (ROI) for in vitro cell culture

For each 3D image, the pixel intensity values were plotted as a histogram to manually determine a heuristic threshold to remove the majority of pixels with low signal to noise ratio that could be attributed to background noise. All pixels above the threshold were used to define the location of the ROI (Fig. 1DE). This approach, however, did not guarantee continuity of the ROI, because regions of low signal to noise intensity could produce holes within the ROI boundaries. To resolve this issue we used morphological closing techniques to adjust the ROI boundaries, eliminate holes and low intensity regions around the border, and produce a homogeneous 3D globule shaped ROI. The degree of morphological erosion was set empirically to ensure that any background signals were removed. All brightness variations within the image (largely dependent on the depth of focus) were corrected using histogram equalization inside the ROI.

### Masking the Region of Interest for explants

Automated ROI selection in retinal explants was complicated by shadows cast by superficial retinal layers (mostly from blood vessels). A semi-automatic approach was therefore used in which multiple ROIs within the IPL were first selected manually using a custom designed graphical user interface (GUI). These selections were then adjusted automatically as for the cell cultures.

### Feature selection

To reduce data dimensionality each ROI was parameterized using 65 features. The entropy, range, and standard deviation were calculated for every pixel of the 3D image and for each of three matrices mean, median, entropy and standard deviation, were calculated across the entire ROI; the values were normalised according to the grey scale resolution of the original image (for example, for the 8 bit image the entropy value was divided by 8 and standard deviation multiplied by 2) thereby producing the first 12 features. The 3^rd^ and 4^th^ moments of pixel intensity distribution were averaged across the ROI adding 2 more features. Three co-occurrence matrices were computed with an offset of 5 pixels (size of a single cell) along one of three x, y, and z coordinates. For each of the co-occurrence matrices the contrast, correlation coefficient, energy and homogeneity were averaged across the entire ROI, producing the next 12 features. The gray scale image was then transformed into a binary black and white image and the number and size of contiguous black patches in the image (referred to as particles) was estimated. The data were quantified using 12 bins histogram, where each bin represented the number of particle of a given size. The value of each bin was taken as an additional feature. The histogram bin was then doubled in size, resulting in 6 bins histogram (6 features), and doubled again - 3 bins histogram (3 features), bringing the number of granulometry features to 21. The directional wavelet analysis was performed [50], [51] along the x and y axes, and at 45 degree between the x and y axis; for each of the directions mean and standard deviation of coefficients for the three smallest scales were calculated, adding a further 18 features.

### Feature Analyses

Each class of data (e.g. apoptotic vs control) was represented by a Gaussian Mixture Model (GMM). This approach has the advantage of allowing for the presence of subpopulations within the class (in our case the number of subpopulations was set to 4) without any a priori knowledge of their parameters. The best model parameters were determined in accordance with Akaike Information Criteria [52] (AIC):
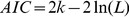
(1)where k is the number of subpopulations, and L is the likelihood function characterising the fit of the model to the training data.

The validation stage consisted of presenting a “new” image to be classified into one of the existing classes based on the Mahalanobis distance [17]:
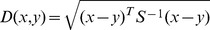
(2)where x is a point in feature space representing a new image and y is a point representing the centre of a cluster of a given class; S is the covariance matrix of the cluster. The new image was assigned to a class separated by the minimal distance. The cross-validation technique (“leave-one-out”) was used to assess the accuracy of this classification. For each step of cross-validation most of the data (excluding one subset) were used for the training procedure (e.g. finding parameters for each GMM). The excluded subset was then used for model validation, e.g. all images of this subset were classified (correctly or incorrectly) to one of the classes learned from the training set. Multiple rounds of cross-validation were performed, and the percentage of correctly classified images was estimated.

To visualise multidimensional data a Sammon [18] projection along the axis with the largest variance was used. Since the Mahalanobis distance [17] is only defined for two points within the same class/cluster, the distance between two points from two different clusters was calculated as following:
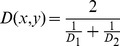
(3)where D_1_ is Mahalanobis distance computed using the covariance matrix of the first cluster and D_2_ is Mahalanobis distance computed using the covariance matrix of the second cluster. The Centre of Gravity (COG) plots were produced by finding the mean of each cluster; the confidence intervals were computed, assuming normal data distribution.
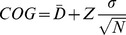
(4)Where 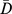 is an average distance, Z is the upper critical value for the standard normal distribution (1.96 for 95% confidence interval), N is a number of points (images used for validation), and 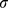 is the standard deviation of distance.

Feature Reduction. Feature reduction [20] helps to further decrease dimensionality and speed up the learning process. The feature reduction was performed using sequential forward search and an additional cross-validation (“leave-one-out”) procedure over each training set. In practice, for each step of the analysis one data subset was chosen for validation (classification) and the rest of the data were used for training as before. However, the training set was also subdivided. One data subset from the training set was used to select the most relevant features, and the rest of the training data were used to find GMM parameters as a function of selected feature set. The procedure was repeated for every subset from the training set and the multiple sets of “optimal” features were produced. Due to the limited set of training data “union” of all the relevant features was performed. It is important to note that classification was performed on the data subset that was different and completely independent from the training set. For the larger set of training data, the optimal feature set was formally defined based on the binary nature of classification results. The classification error p can be described by the Bernoulli distribution (a form of exponential distribution), thus the optimal number of features k can be estimated using Bayesian Information Criterion (BIC) [21]:
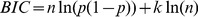
(5)where n is the number of features. During training, all features were sorted based on their classification contribution and the resulting set formed by adding classification features one by one, starting from the most relevant feature, while evaluating BIC after each addition. The minimum BIC value corresponded to the most appropriate feature set.

### Identifying the optical signature of apoptotic RGC-5 cells 24 h after treatment with staurosporine

For the first experiment, data were collected from 20 apoptotic and 20 control RGC-5 coverslip cultures with each coverslip imaged at 10 different locations. The quality of all images was visually inspected: images that were out of focus, had air bubbles in the cell media or a build-up of moisture on the cover slips were discarded. The remaining 120 control and 120 apoptotic 3D images (also referred to as images or image stacks) were selected for analyses. After initial image pre-processing, a fully automated approach was used to locate the cells within the region of interest (ROI).

A set of 65 parameters/features that described each ROI in compact form was then defined. We used the cross-validation (“leave-one-out”) technique to evaluate the statistical accuracy of the classification. Data were divided into subsets, containing 10 to 20 image stacks according to the imaging session (to minimise variability due to the manual adjustment of parameters such as light polarisation during acquisition).

### Identifying the optical signature of early apoptosis in RGC-5 cells

Data were collected from 34 separate RGC-5 cultures undergoing apoptosis, restricting our analyses to the first hour after application of staurosporine. Some of the images were discarded after initial visual inspection due to poor quality, leaving 28 cultures, that were imaged at single location every 10 min from 10 to 60 min post staurosporine treatment (168 image stacks in total, divided into 6 subsets according to the imaging session).

### Identifying the optical signature of early apoptosis in ex vivo murine retinal explants

The 13 retinas were analysed and 10 ROIs (128×64×64 pixels) were defined within the IPL of each retina. This produced 130 image-stacks for each time point; the data were further divided into 5 subsets.
